# Epidemiologic characteristics of outbreaks of three norovirus genotypes (GII.2, GII.17 and GII.4 Sydney) in Guangzhou, China, from 2012 to 2018

**DOI:** 10.1017/S0950268819000992

**Published:** 2019-05-29

**Authors:** H. Wang, D. H. Wang, C. Chen, Y. Lu, M. X. Li, T. G. Li, Z. B. Zhang, Z. C. Yang

**Affiliations:** Guangzhou Centre for Diseases Control and Prevention, Guangzhou, China

**Keywords:** Genotype, noroviruses, outbreak

## Abstract

To compare the epidemiologic features (e.g. settings and transmission mode) and patient clinical characteristics associated with outbreaks of different norovirus (Nov) strains, we retrospectively analysed data of Nov outbreaks occurring in Guangzhou, China from 2012 to 2018. The results suggested that outbreaks of Nov GII.2, GII.17 and GII.4 Sydney exhibited different outbreak settings, transmission modes and symptoms. GII.2 outbreaks mainly occurred in kindergartens, elementary and high schools and were transmitted mainly through person-to-person contact. By contrast, GII.4 Sydney outbreaks frequently occurred in colleges and were primarily associated with foodborne transmission. Cases from GII.2 and GII.17 outbreaks reported vomiting more frequently than those from outbreaks associated with GII.4 Sydney.

Noroviruses (NoVs) are leading causes of gastroenteritis worldwide and can be transmitted by different routes (e.g. foodborne, person-to-person, waterborne and environmental transmission) [1]. NoVs were responsible for 47% of all gastroenteritis outbreaks of known aetiology reported in China from 2006 to 2012 [2]. NoVs can be genetically divided into seven genotypes (GI–GVII), with GI and GII strains responsible for most human diseases. In March 2012, a new NoV strain called GII.4 Sydney was identified in Australia and has since caused acute gastroenteritis outbreaks worldwide [3, 4]. In January 2013, GII.4 Sydney became the predominant circulating NoV strain and caused massive outbreaks in Guangzhou, prompting public concern regarding potential increases in the incidence and severity of outbreaks [5]. Subsequently, during the winters of 2014–2015 and 2016–2017, the NoV genotypes GII.17 and GII.2 emerged and caused increased outbreak activity in Guangzhou and other districts. To compare the epidemiologic features (e.g. settings and transmission mode) and patient clinical characteristics associated with outbreaks of different NoV strains, we retrospectively analysed data of NoV outbreaks occurring in Guangzhou from 2012 to 2018.

## Norovirus surveillance and analyses

Since 2012, the Guangzhou Centres for Disease Control and Prevention have conducted surveillance for NoV outbreaks through the Notifiable Infectious Disease Reporting Information System (NIDRS). NIDRS is a comprehensive reporting system for all notifiable infectious diseases in China. An outbreak, defined as ⩾20 acute gastroenteritis cases with diarrhoea (⩾3 episodes within 24 h) and/or vomiting (⩾2 episodes within 24 h) associated with a common source and occurring within 7 days, must be reported to NIDRS within 24 h. For each outbreak, cases were interviewed face-to-face using a semi-structured questionnaire by trained public health officers to identify the suspected transmission route. Information collected included demographic characteristics, illness onset, symptoms, duration of illness, food and drink consumption and contact history with suspected cases. Samples from each outbreak were first tested for NoVs. Molecular typing of NoV strains was performed for NoV-positive specimens using standardised laboratory protocols for reverse transcription PCR and sequence analysis [6]. Descriptive statistics were used to analyse the epidemiologic and clinical characteristics of outbreaks and patients. The *χ*^2^, Fisher's exact or Kruskal–Wallis (K-W) tests were used to compare differences among strains. *P*-values were derived from two-tailed tests and significant was assumed for *P* < 0.05. All statistical analyses were performed using SPSS 21.0 (SPSS Inc., Chicago, IL, USA).

## Key findings

**Fig. 1.**
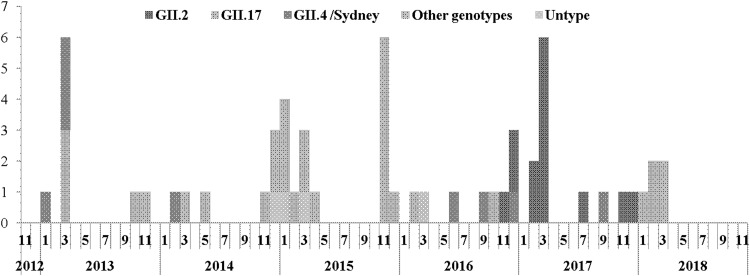
Number of reported norovirus outbreaks in Guangzhou, Southern China, from 2012 to 2018.

From November 2012 to November 2018, 58 NoV outbreaks were reported involving 4755 clinical cases. NoV outbreaks were highly seasonal in Guangzhou with most (86.2%) outbreaks occurring from November to March (Fig. 1). Sequence data were available for 54 outbreaks, and the most commonly reported causative strains were GII.17 (*n* = 17 (31.5%)), GII.2 (*n* = 14 (25.9%)) and GII.4 Sydney (*n* = 9 (16.7%)). Less commonly, outbreaks involved multiple NoV genotypes (*n* = 4 (7.4%)), GII.3 NoVs (*n* = 2 (3.7%)) or GII.6 NoVs (*n* = 2 (3.7%)). NoV GII.12, GII.14, GI.6, GI.7 were each responsible for one outbreak. Of all outbreaks, seven of 13 (53.8%) were caused by GII.17 NoV during the winter of 2014–15 and 11 of 12 (91.7%) were caused by GII.2 NoV during the winter of 2015–16.

The most commonly reported outbreak settings were schools, representing 52 (89.7%) outbreaks. Compared with other strains, GII.2 was reported at higher frequency in kindergartens (21.4%). Outbreaks involving GII.4 Sydney occurred more frequently in colleges/universities (77.8%). In our study, outbreaks transmitted by food handlers were also classified as foodborne. The most commonly identified suspected mode of transmission was foodborne transmission (30 (51.7%) outbreaks) followed by person-to-person transmission (21 (36.2%) outbreaks). Foodborne transmission was more frequently associated with GII.4 Sydney outbreaks than with GII.2 and GII.17 outbreaks. Fisher's test showed that there were significant differences among the GII.4 Sydney, GII.17 and GII.2 strains with respect to outbreak settings (*P* = 0.02) and transmission mode (*P* = 0.02) (Table 1).

**Table 1.** Number and percentage of norovirus gastroenteritis outbreaks by strain, setting and mode of transmission in Guangzhou, from November 2012 to November 2018

Characteristic	Total outbreaks (%)	GII.2 outbreaks (%)	GII.17 outbreaks (%)	GII.4/Sydney outbreaks (%)	*P*^a^
Total	58 (100.0)	14 (100.0)	17 (100.0)	9 (100.0)	
Setting					
School					0.02
Kindergarten	9 (15.5)	3 (21.4)	1 (5.9)	0 (0.0)	
Elementary school	9 (15.5)	4 (28.6)	4 (23.5)	0 (0.0)	
High school	13 (22.4)	4 (28.6)	5 (29.4)	0 (0.0)	
College/university	21 (36.2)	2 (14.3)	4 (23.5)	7 (77.8)	
Restaurant	1 (1.7)	1 (7.1)	0 (0.0)	0 (0.0)	
Industry	4 (6.9)	0 (0.0)	2 (11.8)	2 (22.2)	
Hospital	1 (1.7)	0 (0.0)	1 (5.9)	0 (0.0)	
Mode of transmission					0.02
Person-to-person	21 (36.2)	7 (50.0)	8 (47.1)	0 (0.0)	
Foodborne	30 (51.7)	7 (50.0)	9 (52.9)	7 (77.8)	
Unknown	7 (12.1)	0 (0.0)	0 (0.0)	2 (22.2)	

a Fisher's exact test.

Information on patient age was available for 3491 patients from 48 outbreaks (82.8%). The median age of affected cases was 19 years. The proportion of patients >20 years of age was higher for GII.4 Sydney outbreaks (54.3%), while GII.2 outbreaks were more likely to be observed in cases 5–15 years of age (46.3%). The age distributions of cases differed significantly among NoV strains (*P* < 0.05). The K-W test showed that GII.4 Sydney outbreaks occurred disproportionally among older cases. Cases from GII.2 and GII.17 outbreaks reported vomiting more frequently than those from outbreaks associated with GII.4 Sydney (82.6%, 72.2% and 30.2% of cases, respectively). By contrast, diarrhoea was reported by a higher proportion of cases during GII.4 Sydney outbreaks compared with GII.2 and GII.17 outbreaks (Table 2).

**Table 2.** Number and percentage of patients in outbreaks of acute gastroenteritis attributed to norovirus by sex, age, symptoms and strain in Guangzhou, from November 2012 to November 2018

Characteristics	Total	No. (%) patients linked to outbreaks of norovirus GII.2, GII.17 and GII.4 Sydney				
		GII.2 (%)	GII.17 (%)	GII.4/Sydney (%)	*χ*^2^	*P*^a^
Demographic information^b^	3491 (100.0)	508 (100.0)	939 (100.0)	594 (100.0)		
Sex					30.05	<0.05
Male	1829 (52.4)	267 (52.6)	442 (47.1)	399 (61.0)		
Female	1662 (47.6)	241 (47.4)	497 (52.9)	255 (39.0)		
Age, years					481.15^c^	<0.05
0–4	120 (3.4)	53 (10.4)	11 (1.2)	0 (0.0)		
5–15	665 (19.0)	235 (46.3)	323 (34.4)	0 (0.0)		
16–19	1125 (32.2)	90 (17.7)	228 (24.3)	299 (45.7)		
⩾20	1581 (45.3)	130 (25.6)	377 (40.1)	355 (54.3)		
Patient with symptoms^b^	3969 (100.0)	644 (100.0)	1104 (100.0)	460 (100.0)		
Vomiting	2359 (59.4)	532 (82.6)	797 (72.2)	139 (30.2)	362.82	<0.05
Diarrhoea	2364 (59.6)	233 (36.2)	679 (61.5)	381 (82.8)	248.48	<0.05
Vomiting and diarrhoea	1029 (25.93)	121 (18.8)	372 (33.7)	60 (13.0)		

a *χ*^2^ test.

b Information on demographic characteristics and symptoms was available for 48 (82.8%, 3491 patients) and 53 (91.4%, 3969 patients) of the 58 norovirus outbreaks reported during the study period.

c Kruskal–Wallis test.

## Conclusions

We observed different patterns of transmission modes, outbreak settings, case ages and symptoms for different NoV genotypes. The proportion of outbreaks caused by NoVs peaked during November–March of each year, reflecting the overall winter seasonality of NoV outbreaks. Compared with GII.4 Sydney outbreaks, GII.2 outbreaks more frequently occurred in kindergartens, elementary and high school settings, consistent with the observed age predilection of this strain for younger children. Parra *et al*. also reported that non-GII.4 genotypes infected infants more frequently than other strains, presumably because adults build immunity to different genotypes over time [7]. Before fall 2016, GII.2 was a rare genotype, accounting for <1% of infections in China and 1.5% [8] of globally circulating strains [9]. A lack of pre-existing herd immunity in younger people providing an immunologically naive niche for this new GII.2 strain might contribute to the higher susceptibility of younger children to GII.2 [10, 11]. Moreover, host-related factors or better reporting in schools compared with other settings may have contributed to these results. Further studies are needed to better define the basis for this difference.

The different genotype distributions likely represent the varying stability of strains in different media as well as different contamination methods. This is further evidenced by our finding that transmission mode was significantly correlated with NoV genotype. Interestingly, unlike some previous reports, which concluded that person-to-person transmission was more prevalent in GII.4 NoV outbreaks than non-GII.4 outbreaks [12], we found that a higher proportion of foodborne transmission was associated with GII.4 Sydney outbreaks. By contrast, person-to-person transmission was more frequently associated with GII.2 outbreaks than GII.4 Sydney outbreak. This discrepancy might be related to different outbreak settings. A recently published study suggested that GII.4 NoV outbreaks were significantly more likely to be associated with foodborne transmission in non-healthcare settings [13]. Our results showed that GII.4 Sydney outbreaks were more likely to occur in colleges/universities, while prior studies reported a higher proportion of healthcare-associated outbreaks [4, 14].

Leshem *et al.* reported that diarrhoea was more prevalent in cases infected with GII.4 strains than in those infected with non-GII.4 strains (84.8% *vs.* 75.7%) [4]. In our study, the proportion of cases experiencing diarrhoea during GII.4 Sydney outbreaks was significantly higher than that during GII.17 and GII.2 outbreaks (82.8%, 61.5% and 36.2%, respectively, *P* < 0.05). Moreover, we found that GII.2 outbreaks caused a significantly higher rate of vomiting than GII.4 Sydney and GII.17 outbreaks, similar to the results previously reported for Guangdong Province, China [15].

A major limitation of our analysis was reporting bias. First, only outbreaks with ⩾20 cases were reported, as those involving <20 cases are not included in the NIDRS system of China. Thus, these data may not be representative of country-wide NoV outbreak activity. Second, outbreak identification and reporting may differ across settings, and therefore, genotypes occurring in settings with lower reporting rates may have been under-represented in our analyses. Third, we did not collect data on asymptomatic cases because most of these individuals did not seek medical care.

NoVs are a diverse group of pathogens with variable characteristics. It is urgent to strengthen surveillance for NoV outbreaks to monitor the emergence and impact of new NoV strains and to reduce the burden of NoV disease.
